# Automatic rigid image Fusion of preoperative MR and intraoperative US acquired after craniotomy

**DOI:** 10.1186/s40644-023-00554-x

**Published:** 2023-04-13

**Authors:** Edoardo Mazzucchi, Patrick Hiepe, Max Langhof, Giuseppe La Rocca, Fabrizio Pignotti, Pierluigi Rinaldi, Giovanni Sabatino

**Affiliations:** 1 Unit of Neurosurgery, Mater Olbia Hospital, Olbia, Italy; 2 Institute of Neurosurgery, IRCCS Fondazione Policlinico Universitario Agostino Gemelli, Catholic University, Rome, Italy; 3 Brainlab A.G, München, Germany; 4 Unit of Radiology, Mater Olbia Hospital, Olbia, Italy

**Keywords:** Neuronavigation, Image guided surgery, Ultrasound, Maximal safe resection, Brain tumor, Image co-registration, Image fusion

## Abstract

**Background:**

Neuronavigation of preoperative MRI is limited by several errors. Intraoperative ultrasound (iUS) with navigated probes that provide automatic superposition of pre-operative MRI and iUS and three-dimensional iUS reconstruction may overcome some of these limitations. Aim of the present study is **t**o verify the accuracy of an automatic MRI – iUS fusion algorithm to improve MR-based neuronavigation accuracy.

**Methods:**

An algorithm using Linear Correlation of Linear Combination (LC2)-based similarity metric has been retrospectively evaluated for twelve datasets acquired in patients with brain tumor. A series of landmarks were defined both in MRI and iUS scans. The Target Registration Error (TRE) was determined for each pair of landmarks before and after the automatic Rigid Image Fusion (RIF). The algorithm has been tested on two conditions of the initial image alignment: registration-based fusion (RBF), as given by the navigated ultrasound probe, and different simulated course alignments during convergence test.

**Results:**

Except for one case RIF was successfully applied in all patients considering the RBF as initial alignment. Here, mean TRE after RBF was significantly reduced from 4.03 (± 1.40) mm to (2.08 ± 0.96 mm) (p = 0.002), after RIF. For convergence test, the mean TRE value after initial perturbations was 8.82 (± 0.23) mm which has been reduced to a mean TRE of 2.64 (± 1.20) mm after RIF (p < 0.001).

**Conclusions:**

The integration of an automatic image fusion method for co-registration of pre-operative MRI and iUS data may improve the accuracy in MR-based neuronavigation.

**Supplementary Information:**

The online version contains supplementary material available at 10.1186/s40644-023-00554-x.

## Background

Maximal safe resection is an important prognostic factor for gliomas. [1–3] Neuronavigation of preoperative MRI is the current standard in surgical management of brain tumors. However, neuronavigation systems show limited accuracy due to multiple physical, technical, operational, and biological issues. [4–8] In order to account for non-biological factors limiting the navigation accuracy, e.g. fundamental aspects of optical navigation or operational constraints, such as loose reference systems, head clamps, etc., [9, 10] intraoperative imaging strategies have been developed to enhance and maintain accuracy throughout the procedure. Examples of 3D imaging modalities are intraoperative MRI (iMRI), intraoperative computed tomography (iCT) and 3D intraoperative ultrasound (iUS).

Compared to iCT, iUS imaging is not associated with radiation exposure and more cost-efficient. Furthermore, acquisition time is shorter than for iCT or iMRI allowing to be applied multiple times during surgery, while providing high spatial resolution and reasonable soft tissue contrast. [11–17] Neuronavigation systems enable reconstruction and integration of 3D iUS imaging (Ultrasound Navigation, Brainlab AG, Germany) by means of tracked ultrasound probes and intelligent image reconstruction enabling the surgeon to use this modality in parallel to previously patient-registered MRI data. [18–21] To this end, the iUS software creates a new patient registration, links it to a previously created registration, i.e. registration-based fusion (RBF) and superimposes iUS and MRI scans in the neuronavigation system. RBF requires that both registered scans show a common (physical) coordinate system, which, however, might be unprecise due to above-mentioned factors limiting the navigation accuracy of the preoperatively acquired MRI.

In order to account for non-biological factors compromising the patient registration of the MRI and to enable an automatic update of the patient registration by means of iUS, an image co-registration algorithm to rigidly align preoperative MRI and intraoperative 3D iUS scans has been previously proposed. [22] This method is based on a modal-specific metric to find the optimal registration result [23] and has been successfully applied to retrospective iUS-MRI data before. [24] However, clinical integration of this method on commercially available neuronavigation systems has not been accomplished so far. Therefore, the goal of the present study is to evaluate a similar implementation of an automatic rigid image co-registration algorithm integrated in a CE-certified / FDA-cleared medical device software to enable intraoperative re-registration of MRI derived planning data based on iUS. Since factors related to resection of brain tissue result in loss of the rigid spatial relationship of intracranial anatomies (e.g. CSF leakage, tissue resection etc.) only iUS acquired after craniotomy and right before start of resection have been considered.

## Methods

Twelve consecutive brain tumor patients were included in the present study (Table 1). The local Ethics Committee approved this investigation. All patients signed a written informed consent. For each patient, preoperative MRI and iUS scans were acquired during clinical routine. The image pairs were retrospectively enriched with verification landmarks to determine the accuracy of the automatic rigid fusion algorithm currently under development at Snke OS GmbH (part of Brainlab AG, Germany).

**Table 1:** Demographic and Clinical data. F: female. M: male. IDH: isocitrate dehydrogenase

Patient ID	Sex	Age	Tumor Location	Tumor Histology
1	F	66	Left parietal	Meningioma WHO 2021 grade 1
2	F	50	Left parietal	Meningioma WHO 2021 grade 1
3	M	46	Left frontal	Astrocytoma IDH-mutated WHO 2021 grade 3
4	F	75	Left parieto-occipital	Glioblastoma IDH-wildtype WHO 2021 grade 4
5	M	70	Left occipital	Glioblastoma IDH-wildtype WHO 2021 grade 4
6	F	66	Right temporal	Metastasis (Uterine sarcoma)
7	M	60	Right parietal	Glioblastoma IDH-wildtype WHO 2021 grade 4
8	M	64	Right parietal	Glioblastoma IDH-wildtype WHO 2021 grade 4
9	M	71	Right temporal	Metastasis (Lung microcytoma)
10	F	19	Left frontal	Pilocytic astrocytoma WHO 2021 grade 1
11	M	44	Right frontal	Oligodendroglioma IDH-mutated 1p19q codeleted WHO 2021 grade 3
12	F	54	Left frontal	Metastasis (Melanoma)

### Neuronavigation Protocol

The MRI protocol preoperatively acquired for neuronavigation the day before surgery was:

- T1 (voxel size = 1*1*1 mm, FOV = 24 cm, slice thickness = 1 mm, TA = 3 min).
- T2 (voxel size = 0.8*0.9*2.6 mm, FOV = 26 cm, slice thickness = 2.6 mm, TA = 5 min).
- FLAIR (voxel size = 0.9*1.2*4 mm, FOV = 24, slice thickness = 4 mm, TA = 2.23 min).

For neuronavigation, all preoperative MRI scans were rigidly fused, and the 3D T1-weighted MRI was registered to an iCT acquired for patient registration by means of CT-based automatic image registration (Cranial Navigation with Automatic Image Registration, Brainlab AG, Germany). An iUS scan is conducted before dural opening using a dedicated machine (bk5000, BK Medical Holding Company, GE Healthcare, United States). The echograph is integrated with the neuronavigation system (Ultrasound Navigation, Brainlab AG, Germany) which provides user-guided continuous, optically tracked iUS sampling and can be used to reconstruct a patient-registered 3D iUS dataset for neuronavigation. [19, 21] However, changes to the registration system and thus inconsistencies between the patient-registered MR and separately registered iUS scan may affect the superposition of both modalities, i.e. RBF, and re-registration using an automatic image co-registration, i.e. rigid image fusion (RIF), may address this ambiguity by providing an update to the initial registration of the preoperative MRI.

### Automatic rigid image Fusion

The prototype implementation of the automatic MRI-iUS rigid image fusion algorithm retrospectively evaluated in this work (release process is ongoing during preparation of the manuscript) was inspired by earlier works [22, 23] and has been previously successfully applied to representative MR-US registration scenarios. [24] In essence, this rigid co-registration algorithm applies Linear Correlation of Linear Combination (LC2)-based similarity metric which exhibits local invariance to how much two channels of information contribute to an US image, i.e. correlation of the US image with both the MRI intensity values and its spatial gradient magnitude, and allows for fully automatic MR-US registration in the matter of seconds. [22]

### Landmark Definition

To evaluate image co-registration accuracy of the proposed image fusion algorithm, landmark pairs were retrospectively determined for reliably identifiable intra-cranial anatomical structures of the MR and the US scan. Brain shift may affect soft and hard tissues in different ways. In order to be representative of the real fusion in each patient, landmarks were chosen on both rigid (e.g. falx, skull base) and deformable structures (ventricles, sulci, vessels) at different distance from the craniotomy. Moreover, it should be easy to identify them both on US and MRI images.

**Fig. 1:**
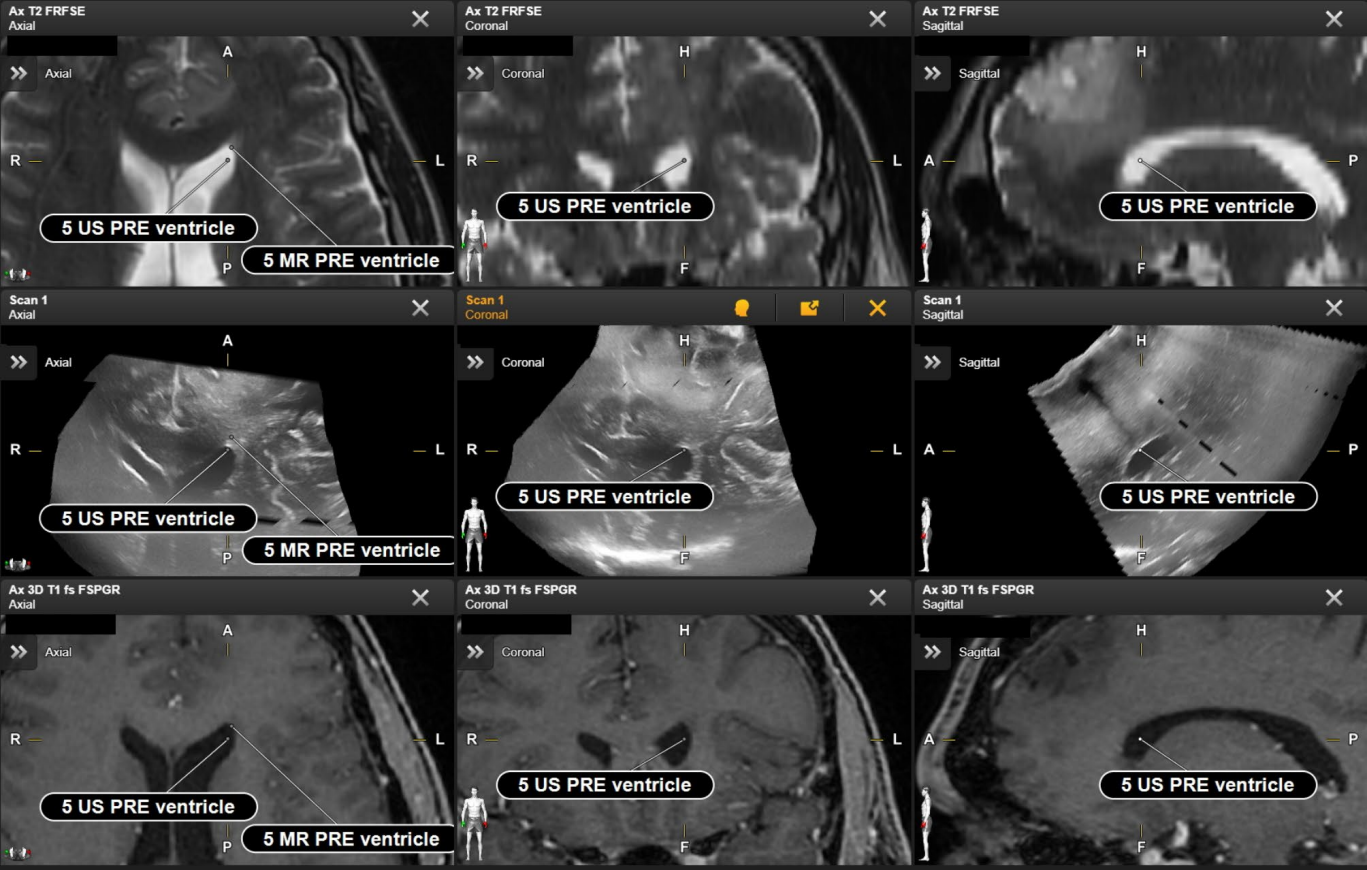
Clinically determined landmarks for a representative patient (Patient 3) with a non-contrast enhancing glioma. The landmarks are displayed in axial, coronal and sagittal projections in T2 (top row), iUS (middle row) and T1 with contrast images (lower row) simultaneously, based on a registration-based fusion (RBF) of the preoperative MR and intraoperatively obtained iUS scans before dura opening. A spatial shift of anatomical structures, e.g. the lateral ventricle, can be observed based on the RBF data

**Fig. 2:**
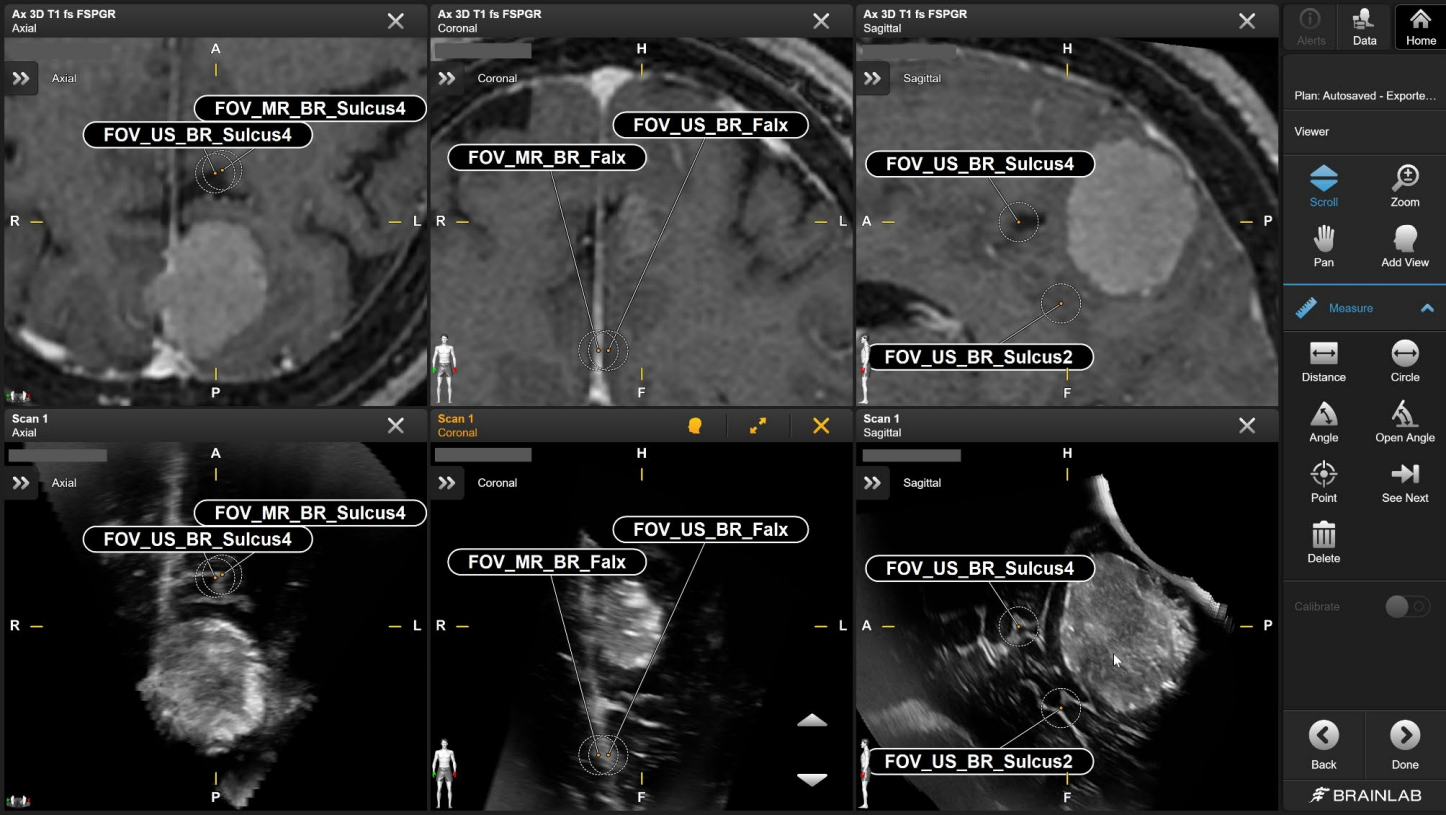
Representative landmarks for patient (Patient 2) showing a lesion in the occipital lobe. A spatial shift of anatomical structures, e.g. the falx, can be observed based on the RBF MR-US data

A multi-staged approach has been chosen to ensure high landmark precision and address interrater variability. First, a clinical expert labelled relevant structures as initial proposal. Second, proposed landmarks were technically verified (i.e. labelling of the right datasets, following the naming convention) and refined if needed, and, lastly, clinically validated by the medical expert. All clinically confirmed landmarks have then been used for automated batch-processing to apply RIF and subsequently measure the Euclidean distances for each landmark pair. Examples of different pairs of landmarks are shown in Figs. 1 and 2.

### Quantitative performance test

The reliability of the proposed fusion algorithm was determined in two steps. First, the RBF was used to calculate the automatic RIF. Second, RIF was calculated for a broad range of initial image pre-alignments i.e. clinically representative capture range of RBFs. This allows to investigate the performance of the proposed algorithm for real-world clinical cases and provided information on the method’s sensitivity to different initial alignments. Therefore, Euclidean distance between a landmark defined in preoperative MR image space and the equivalent landmark identified in the US scan was determined, the so-called Target Registration Error (TRE). TREs are averaged across available landmark pairs in each fusion pair and measured before and after fusing the images automatically.

To consider different initial alignment of the datasets, a convergence test was performed. This test was inspired by previous work. [22] The basic principle is to use landmark-based fusion parameters – as derived from an automatic rigid registration using landmark coordinates only, landmark-based fusion (LBF) – to provide the initial alignment between the MR and US scan. This alignment is then perturbed by random rotations and translations, and an automatic fusion is calculated at each new perturbed position. Measuring the mean TRE at the perturbed position gives a measure of the initial image alignment. Measuring the mean TRE after automatic fusion gives a measure of the algorithm accuracy. This approach has several advantages:

- Increased testing power, since many fusions per image pair can be computed, each with slightly different initial alignment.
- A range of possible initial alignments that includes and goes beyond what is present in the original datasets is covered.
- It allows to determine sensitivity to initial alignment.

The following perturbation parameters were selected to cover a range of possible and realistic initial alignments between the image pairs:

- Rotation range: 0° – 10°.
- Translation range: 0–40 mm.
- Number of perturbations per image pair: 100.

Once the input data is defined (image pairs + landmarks), the test is performed in a fully automated manner within the testing infrastructure at Snke OS. This includes (1) computing the landmark-based initial alignment, (2) perturbing this initial alignment, (3) computing the automatic image-based fusion from the perturbed position, (4) computation of the mean TRE values averaged across patient-individual landmarks and (5) quantification of maximum, minimum, mean and median of average TRE for all iterations of the convergence test per patient dateset.

## Results

**Fig. 3:**
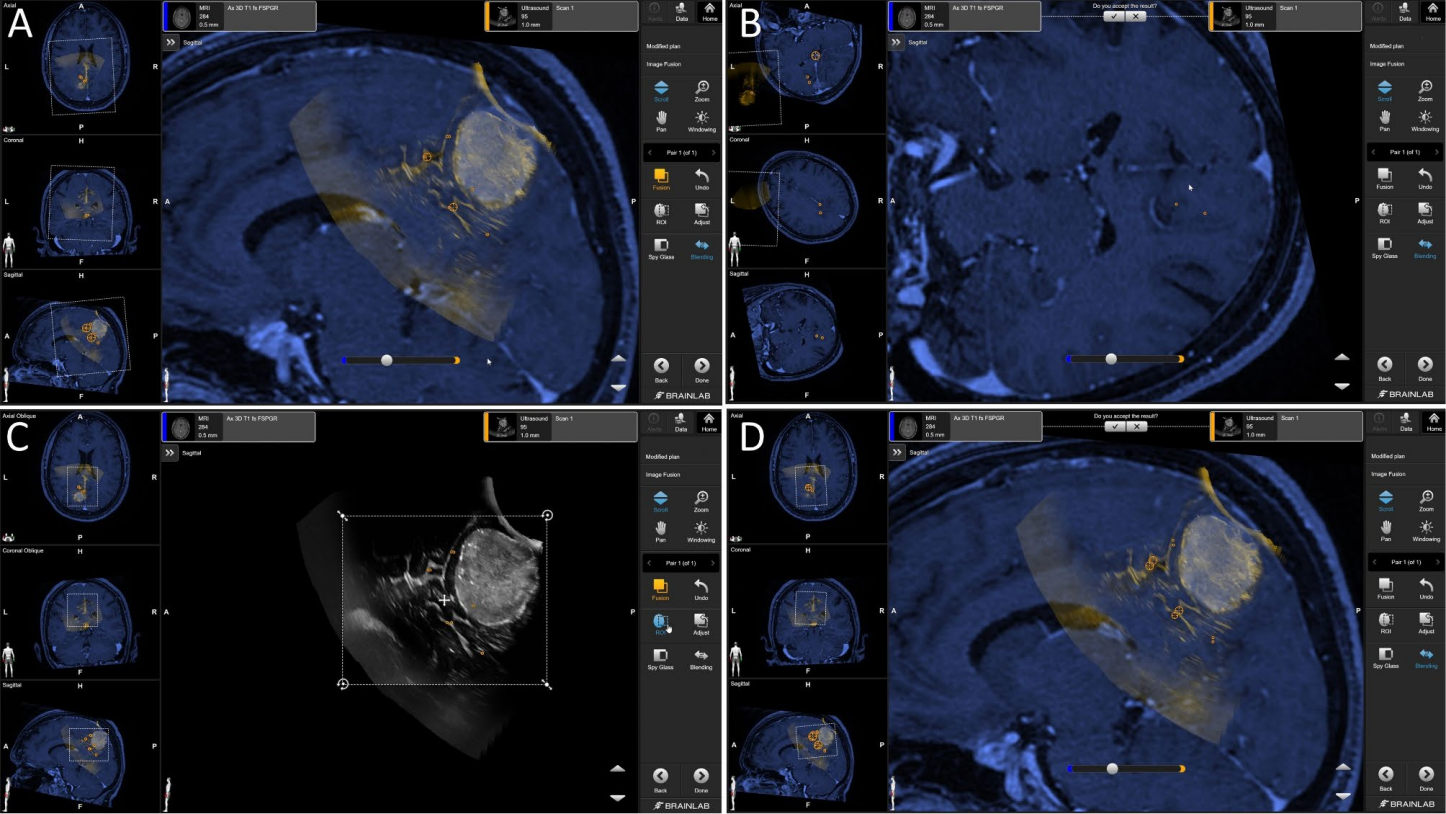
Patient dataset excluded from quantitative evaluation of the automatic image fusion algorithm. This dataset showed initial misalignment of the RBF (**A**) and the algorithm using a default fusion region of interest (ROI), which is determined based on the field of view of the ultrasound scan was not able to align both datasets in fully automated manner (**B**). After manual definition of the fusion ROI – focusing on the clinical target and some deep anatomical structures serving as references for the RIF (**C**) – the proposed method was able to provide a highly accurate co-registration result (**D**)

**Fig. 4:**
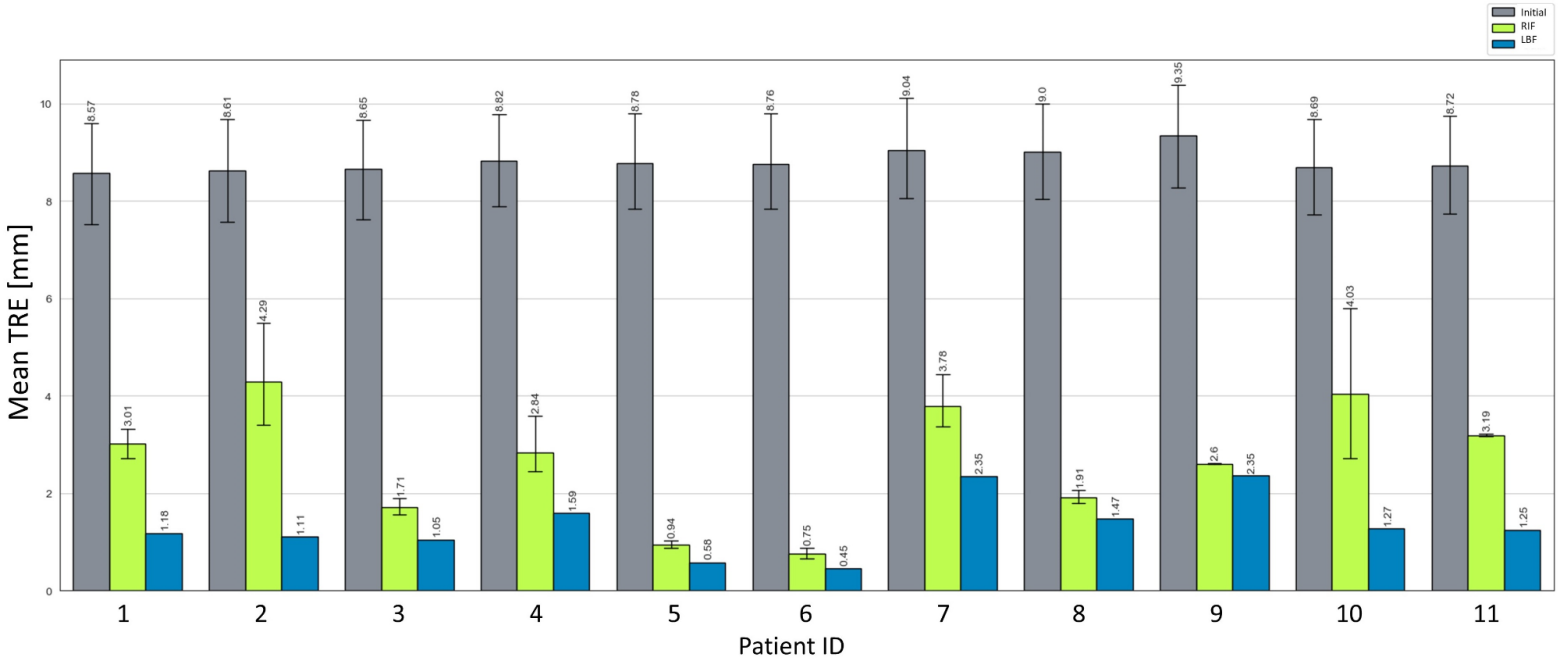
Patient individual mean Target Registration Error (TRE in mm) determined by means of convergence test simulating a set of perturbations of the initial rigid alignment of each MR-US pair (TRE initial), measuring the spatial registration after proposed automatic Rigid Image Fusion (RIF), and in light of best possible fusion accuracy considering determined landmarks as reference only (Landmark Based Fusion, LBF).

Except for one patient (Patient 2), all calculations could be successfully performed without the need of any manual interaction (in terms adjusting the ROI used for image fusion). The patient requiring manual adjustment of the fusion ROI was excluded from further quantitative analysis (and was qualitatively assessed in Fig. 3). The results for RBF quantitation and subsequently calculated RIFs are given in Table 2. On average, a mean TRE of RBF of 4.03 (± 1.40) mm was determined, whereas RIF showed a TRE of 2.08 (± 0.96) mm representing a significant reduction of the registration error (*p* = 0.002). In concordance, the convergence test simulated alignment, thus mean initial TRE values was 8.82 (± 0.23) mm as given in Table 3. The mean TRE for RIF after the convergence test was 2.64 (± 1.20) mm, with a significant improvement of accuracy (*p* < 0.001). Results of the convergence test are illustrated in Fig. 4.

**Table 2:** Patient-individual mean Target Registration Error (TRE in mm) after Landmark Based Fusion (LBF), registration based fusions (RBF) and the proposed Rigid Image Fusion (RIF), latter performed based on RBF.

Patient ID	LBF	RBF	RIF (after RBF)
1	1.18	7.38	2.23
3	1.11	3.78	3.25
4	1.05	3.04	1.16
5	1.59	2.60	2.42
6	0.58	3.67	0.88
7	0.45	2.23	0.62
8	2.35	5.23	3.25
9	1.47	3.86	1.68
10	2.35	4.37	2.60
11	1.27	3.75	1.56
12	1.25	4.37	3.17
Mean	1.33	4.03	2.08
Standard deviation	0.61	1.40	0.96

**Table 3:** Convergence test for Rigid Image Fusion (RIF) yielding higher mean Target Registration Error (TRE in mm) values after perturbation (i.e. initial TRE in mm) compared to RBF and the proposed method shows significantly reduced values after Rigid Image Fusion (RIF) (p < 0.001)

Patient ID	Intial	RIF
1	8.57	3.01
3	8.61	4.29
4	8.65	1.71
5	8.82	2.84
6	8.78	0.94
7	8.76	0.75
8	9.04	3.78
9	9.00	1.91
10	9.35	2.60
11	8.69	4.03
12	8.72	3.19
Mean	8.82	2.64
Standard deviation	0.23	1.20

To interpret the results in terms of reliability of the landmark definition, LBF has been quantified additionally yielding a mean TRE of 1.33 (± 0.61) mm. This indicates that the landmarks are associated with a certain imprecision limiting the theoretically assessable accuracy of RIF.

## Discussion

Intraoperative orientation and recognition both of tumor border and of eloquent structures is fundamental to achieve the best oncological and functional outcome. Neuronavigation of pre-operative imaging may be inaccurate due to various technical and physiological sources of error. The integration of intraoperative imaging and multiple monitoring techniques aims at reducing the uncertainty of neuronavigation, thus improving both safety and efficacy of resection [14, 25–28].

Intraoperative MRI represents the state of the art in neuro-oncological surgery, but it is available only in few neurosurgical centers. Intraoperative CT has been recently established to automatically register a preoperatively obtained MRI dataset by means of tracked iCT scanning and automatic multi-modal MR-CT rigid image fusion, yielding a patient registration accuracy below 1.5 mm. [29–31] It is less expensive and faster than iMRI, but it produces lower quality images and requires the exposure of the patient to radiation. Ultrasound is the cheapest and fastest of the three available intraoperative imaging techniques, but interpretation of the acquired images is more difficult. Neuronavigation systems that allow immediate superimposition of iUS images with (typically more familiar) MRI scans may facilitate identification both of tumor and of eloquent cortical and subcortical areas. This fusion relies on the initial registration of the patient; it is thus affected by the same sources of error described for neuronavigation. [7] In the present study the mean TRE of RBF, determined as initial superimposition accuracy of pre-operative MRI and 3D iUS (automatically provided by the commercially available software used intraoperatively) was 4.03 mm with a maximum patient individual mean TRE of 7.38 mm. Interestingly, the initial registration was conducted by means of iCT-based automatic image registration that was previously reported to represent a very accurate method. [29, 32] Therefore, the navigation errors registered in this work are most likely caused by operational, technical effects, e.g. mechanical forces applied during craniotomy or loose reference marker adapters.

Prada et al. [33] described a workflow for intraoperative adjustment of neuronavigation based on iUS. It is an efficient method, highly dependent on experience of the user, with a widely variable processing time. Here, preliminary results of a new method for automatic adjustment of MRI-iUS fusion are presented to evaluate the performance in terms of accuracy and workflow efficiency (i.e. reduction in the processing time without any user dependence). It is (semi-) automatically integrated in the navigation workflow to improve the quality of initial registration within a few seconds (see Video 1, showing that a user interface application needs to be started and approved upon accuracy review). This may facilitate, for example, correction of errors due to inaccurate registration (without the need of iCT [29]) or of minimal movements of the reference array or of the head.

This pilot investigation has been performed considering iUS data acquired before dural opening only, and therefore on data which reflect intra-individually the same anatomical situation of pre-operative MRI. Future studies are required to evaluate the capabilities of the proposed method to apply it to compensate for brain shift which occurs during surgical resection.

The TRE determined in this work is in line with previously described methods for fusion of MRI and US images. [22, 34–36] However, TRE measurements are generally prone to systematic and random e.g. subjective errors, such as due to limited spatial resolution and non-rigid modifications to intracranial anatomies or uncertainties present during the process of recognition of same anatomical structures in both imaging modalities (MRI and iUS), respectively. Brain deformations produced by the convex probe during US scanning (with the probe gently pushed on the dura or parenchyma to maximize the area of contact), may limit rigid fusion and result in biased TRE measurements (probably more prominent for superficial and softer structures while mostly absent for deep / rigid structures). As a consequence, the TRE cannot be equal to zero (even for the most advanced algorithm). In our case series the LBF, which is the fusion acquired using landmark coordinate information only, thus representing the lowest possible TRE, was on average 1.33 mm. Therefore, it can be argued that the landmark definition is limited to this kind of uncertainty in terms of spatial localization and that a TRE of approximately 2 mm for the RIF represents an optimal co-registration result.

### Limitations

In one case (patient 2) automatic RIF failed and a manual definition of the ROI used for image fusion calculation was necessary to successfully achieve a reasonable RIF result (please see Fig. 3). The effect of registration artifacts is a consequence of a statistically possible error of the fusion algorithm (and in the nature of the algorithm relying on statistical principles to optimize similarities) which may occasionally result in iUS scans co-registered with MRI datasets in other parts of the brain (or even outside the skull). In this case, RIF resulted in a co-registration error that was evident, even for an inexperienced user, so it would not have been misleading in the clinical practice. An optimal RIF (Fig. 3) was achieved after manual constraint of the ROI which is per definition initially defined according to the dimensions of the acquired 3D iUS scan. Nevertheless, such artefacts reinforce the awareness that the surgeon must verify the image fusion result. The occurrence of this error will, however, be object of specific tests to further refine the proposed method.

The present article provides preliminary results in a small cohort of patients. Future studies are deemed necessary to evaluate the clinical value of the method prospectively, in a larger group of patients and at different phases of surgery, to verify its efficacy in correcting the brain shift that occurs during resection.

## Conclusions

The integration of an algorithm for automatic fusion of pre-operative MRI and iUS may improve the accuracy of target registration in neuronavigation for brain tumor. Future studies will evaluate its efficacy in clinical practice in a larger cohort of patient.

## Electronic supplementary material

Below is the link to the electronic supplementary material.

**Video 1**: Screen capture video showing the user interface application that has to be launched and approved to provide the automatic rigid image fusion

## Data Availability

The data that support the findings of this study are available from the corresponding author, upon reasonable request.
